# Spiritual Orientation and Religious Coping Styles of Mothers whose Babies were Hospitalized in a Neonatal Intensive Care Unit in Türkiye: Relationship with Stress Levels and Parental Beliefs

**DOI:** 10.1007/s10943-025-02316-4

**Published:** 2025-04-24

**Authors:** Dilek Küçük Alemdar, Muhammet Bulut, Dilara Cengiz, Özge Eren

**Affiliations:** 1 Department of Nursing, Ordu University Health Science Faculty, Ordu, Turkey; 2 Department of Pediatrics, Faculty of Medicine, Giresun University, Giresun, Turkey; 3 Department of NICU, Giresun University Women and Child Education and Research Hospital, Giresun, Turkey; 4 Quality Directorate Unit, Giresun University Women and Child Education and Research Hospital, Giresun, Turkey

**Keywords:** NICU, Coping, Parental belief, Religious, Spiritual orientation, Stress

## Abstract

This descriptive, correlational study examined the relationship between spiritual orientations, religious coping styles, stress, and parental beliefs in mothers with infants in the neonatal intensive care unit (NICU). The study included 120 mothers whose infants were treated in a hospital NICU. Data were collected using the Mother and Infant Descriptive Information Form, Parental Stressor Scale: NICU (PSS:NICU), NICU Parent Belief Scale (PBS), Spiritual Orientation Scale (SOS), and Religious Coping Styles Scale (RCSS). There was a significant correlation between SOS and PBS (*β* = 0.364; *t* = 4.251; *p* < 0.001) and explained 13% of the variance (*R*^2^ = 0.133). It was also found that there was a significant correlation between positive religious coping styles and PBS (*β* = 0.256; *t* = 2.873; *p* = 0.005) and explained 6% of the variance (*R*^2^ = 0.065). However, no significant correlation was found between Negative Religious Coping Styles and PSS:NICU (*p* = 0.339). These findings emphasize the relationship between spiritual orientation, religious coping styles, parental stress and beliefs.

## Introduction

The transition from pregnancy to parenthood is both an exciting and stressful milestone for parents (Refaeli et al., 2024). While parents who give birth to a healthy baby can manage this process successfully, newborns who require hospitalization and intensive care treatment for various reasons after birth may lead to serious emotional difficulties in all family members, especially mothers (Erdeve et al., 2008; London et al., 2007). Treatment and follow-up in the NICU may be necessary for newborns due to perinatal complications, respiratory distress, inadequate nutrition, thermoregulation problems, congenital anomalies, and infections, regardless of their birth weight and gestational age (London et al., 2007). Admission of infants to the NICU leads to an intense stress and anxiety process in family members and especially in parents; this process causes complex emotions such as anxiety, fear, shock, panic and guilt (Çelebioğlu, 2004). In this process, the support offered by healthcare professionals to parents, information about the medical condition of the baby and the treatment process, the opportunity to visit the intensive care unit and the active involvement of parents in the care process contribute significantly to the management of the stress experienced by families (Çelebioğlu, 2004; Merey & Şentepe Lokmanoğlu, 2019).

## Background

Recent studies have revealed the effect of spirituality and religious orientations in coping with stress (Akbaş et al., 2019; Fowlie & Mchaffie, 2004). Although religion and spirituality are often used interchangeably, they are actually different concepts (Erdem, 2010; Korja et al., 2009). Although spirituality can be seen as a component of religion, it is too comprehensive to be limited to religious beliefs; it is a universal concept that addresses all biological, physiological and psychological aspects of the individual, gives the power to live and cannot be expressed only through religion (Fowlie & Mchaffie, 2004). It also includes the individual’s relationships with himself/herself and others, his/her place in the universe, his/her relationship with God, and his/her efforts to understand and accept the purpose of life. Spirituality becomes even more important in crisis and stress situations where individuals’ values and beliefs are threatened and they experience feelings of hopelessness, fear and anxiety (Fowlie & Mchaffie, 2004; Özyazıcıoğlu & Güdücü Tüfekci, 2009; Konukbay & Arslan, 2011; Akbaş et al., 2019). Studies show that spiritual orientations make individuals feel hopeful and strong in the face of stressful situations, facilitate coping with stress and acceptance of the disease process, and create positive effects on health by promoting positive health behaviors (Larson & Larson, 2003; Özyazıcıoğlu & Güdücü Tüfekci, 2009; Akbaş et al., 2019).

In a study evaluating the effects of spirituality on physical and mental health, it was found that spiritual coping supports the ability to cope with stress, is effective in controlling anger and resentment, and can play a protective role against depression in addition to increasing the individual’s inner peace (Larson & Larson, 2003). Furthermore, there is evidence that prayer and meditation can improve both physical and psychological parameters (Newberg & Newberg, 2013). Spiritual endeavors reassure individuals and provide balance, support and guidance during critical periods, enabling individuals to hold on to a sense of purpose and meaning even in the midst of challenging events in their lives. Spirituality and religion are a vital source of strength for many people and can play an important role in reducing psychological symptoms and increasing well-being (Corey, 2005).

Religious orientations encourage acceptance instead of denial of negative events that cause stress and interpreting events from a new perspective (Er, 2006; Larson & Larson, 2003; Newberg & Newberg, 2013). According to Pargament (2013), religion interacts with stress coping processes as much as spirituality and can affect these processes both positively and negatively. Approaches such as feeling close to God through worship and prayer in stressful situations, asking for forgiveness, talking to a religious official and considering the problems experienced as a test are among the positive religious coping methods that help control and reduce stress (Corey, 2005; Göcen, 2015; Larson & Larson, 2003). On the other hand, attitudes such as seeing a negative situation that causes stress as a punishment for a mistake, thinking that God has forgotten about him or feeling anger toward God increase stress and are considered as negative religious coping methods (Göcen, 2015; Larson & Larson, 2003). When the literature was examined, it was observed in many studies that positive religious coping methods positively support psychological health, while negative coping methods negatively affect psychological health (Corey, 2005; Göcen, 2015). Many studies on the subject show that positive religious coping methods have positive relationships with psychological health, while negative religious coping methods have negative effects on psychological health (Park et al., 2018; Pichler-Stachl et al., 2019). However, when the research on this process is examined, it is noteworthy that there are a limited number of studies on the spiritual orientations and religious coping styles of mothers whose babies are hospitalized in the NICU (Merey & Şentepe Lokmanoğlu, 2019; Yılmaz & Küçük Alemdar, 2021). In line with this information, our study aims to determine the relationship between spiritual orientations and religious coping styles, stress and parental beliefs of mothers whose infants are hospitalized in NICU.

## Methods

### Setting and Samples

This research is planned as a descriptive and correlational study. The study will be conducted in the NICU of … Obstetrics and Gynecology Training and Research Hospital between 1 March 2022 and 1 March 2023. In addition, the NICU in … Obstetrics and Gynecology Training and Research Hospital, where the babies of the mothers to be included in the study were treated, provides third level service. The unit has a capacity of 28 incubators and 18 nurses are on duty. There are 4 rooms with a total capacity of 1 bed outside the unit for the mothers of the babies hospitalized in the NICU. Accommodation and nutrition requirements of mothers are met here. There is no visit restriction for mothers staying in the mother hotel in the unit. Mothers who do not stay in the mother hotel are allowed to visit 2 times a day. There is no time limit for visiting the unit. The data of the study were collected after the institutional permission and ethics committee permissions are obtained. The population of the study consisted of mothers of newborn babies who received treatment and care for at least 3 days in the NICU of … Obstetrics and Gynecology Training and Research Hospital between March 1, 2022 and March 1, 2023. The sample of the study was determined as a total of 115 mothers by calculating 95% confidence (1-*α*), 95% test power (1-*β*) and *r* = 0.30 correlation value with reference to Cohen (1988) effect sizes by Gpower analysis. Due to the possibility of data loss, the number determined by power analysis was increased and 120 mothers were included in the study sample.

#### Inclusion Criteria

The baby has received treatment for at least 3 days in the NICU, the mother has at least primary education, and the mother has no obstacle to communication.

#### Exclusion Criteria

The mother’s inability to communicate verbally and illiteracy, referral of the baby to an advanced healthcare institution.

## Instruments

### Mother and Infant Introductory Information Form

The "Mother and Infant Introductory Information Form", which was created by the researchers by reviewing the literature, includes demographic data about the mother and the infant, and questions about the prenatal, intrapartum and postnatal periods that are thought to be related to the mother’s spirituality and stress status.

### Spiritual Orientation Scale (SOS)

The Spiritual Orientation Scale was developed by Kasapoğlu (2015) to assess the spiritual orientation of individuals. The scale was designed according to the perspective of belief in a divine power, meaning and search, prayer/meditation, which are accepted as the basic parameters of spirituality. The SOS is a 7-point Likert-type scale consisting of 16 items. This scale is scored from “1 = Strongly Disagree” to “7 = Strongly Agree”. The range of points that can be obtained from the scale varies between 16 and 112. Sample items of the scale; I feel the presence of a higher power in the depths of my soul; My belief in a divine being gives meaning to my life; I feel protected by a higher power; Admiring nature strengthens my spiritual feelings (Appendix 1). The high score obtained from the scale indicates that the level of spiritual orientation is high. Kasapoğlu (2015) determined the Cronbach Alpha reliability coefficient of the scale as 0.87 and the test–retest value as 0.84.

### Religious Coping Styles Scale (RCSS)

Religious Coping Styles Scale is a scale developed by Pargament et al. (1998) based on the relationship between coping, religious coping and a series of psychological data of three groups with different life events. In this scale, there are 7 items related to Positive Religious Coping Style (items 1, 2, 3, 4, 5, 6, 7) and 3 items related to Negative Religious Coping Style (items 8, 9, 10). The Religious Coping Scale was adapted to the Turkish society by Ekşi in 2016 by performing validity and reliability. The scale is a 4-point Likert-type scale. It is scored as almost never = 1, occasionally = 2, moderately = 3, frequently = 4. In the study of Ekşi and Sayın (2016), the Cronbach alpha internal consistency coefficient of the scale was calculated as 0.91 for the positive religious coping subscale and 0.86 for the negative religious coping subscale.

### Parent Stress Scale: Neonatal Intensive Care Unit (PSS: NICU)

The experience of having a newborn in the neonatal intensive care unit (NICU) can be overwhelming for parents. It was developed by Miles et al. (1993) to determine the perception levels of stressors arising from the physical and psychosocial environment in the NICU by parents. This scale has been widely used in research to assess parental stress and its contributing factors. The validity and reliability study of the scale for our country was carried out by Turan and Başbakkal (Turan & Basbakkal, 2006). Parents are asked to rate the level of stress caused by each of the factors in the scale from one (no stressor) to five (extreme stressor). Factors with a mean score between 3.0 and 3.40 are considered moderately stressful, and factors with a mean score > 3.40 are considered very stressful. Stress factors are analyzed in 3 subgroups. The first is the "Images and Sounds" subgroup, which includes factors such as the presence of equipment on the baby or in the intensive care unit, sounds arising from them and the environment. The second is the “Appearance and Behavior of the Baby” subgroup, and the third is the factors related to the “Role of the Parents”. While filling in the statements such as the presence of tubes and other equipment on or near the baby, sudden change in the baby’s color or the baby’s breathing stopping, mothers whose babies did not have these symptoms did not mark these items.

### NICU-Parental Beliefs Scale (PBS)

This scale developed by Melnyk et al. (2014) has three sub-dimensions (Parenting Role Belief, Parent-Infant Interaction and NICU Knowledge) and consists of a total of 18 items (Melnyk et al., 2014). Each item is scored from 1 (strongly disagree) to 5 (strongly agree). The lowest score obtained from the scale is 18 and the highest score is 90. Sample items of the scale; I feel comfortable in caring for my baby in the NICU.; I am sure about what things I can do to best help my baby get through the NICU experience.; I know the best times to communicate with or interact with my baby.; 16. I am confident in asking the doctors and nurses questions about my baby’s medical condition. (Appendix 2). The higher score obtained from the scale indicates that mothers and fathers have more positive parenting beliefs. The Turkish validity and reliability study of the scale was conducted by Özdemir and Alemdar in 2019 (Özdemir & Alemdar, 2019).

### Data Collection

Mothers were informed about the study before data collection. Then, the data collection forms were delivered in a sealed envelope to the mothers whose babies were hospitalized in the NICU, who voluntarily agreed to participate in the study and who were eligible for the research criteria, when they came to the first visit of the newborn baby. Then, the data collection forms were filled in by the mothers in the waiting room under the supervision of the researcher. Between the data collection dates, all mothers who met the study criteria agreed to participate in the study and completed the questionnaires. It took an average of 15–20 min for the mothers to fill in the data collection forms.

## Ethical Considerations

Written informed consent was obtained from all participants. The study was conducted in accordance with the Declaration of Helsinki and Ethics Committee approval was obtained from the Clinical Research Ethics Committee of the Ordu University. Approval was obtained from the Provincial Health Directorate. The mothers who participated in the study were informed that the study was voluntary and that they could leave the study at any time and the "principle of voluntariness" was complied with. The "confidentiality principle" was complied with by informing the mothers participating in the study that their information would not be shared with other persons and institutions, the results obtained from the research would be used for scientific purposes and personal information would not be disclosed (approval number: 2022/1924).

## Statistical Analysis

The data were analyzed with the statistical package program on the computer. The data to be obtained as a result of the research were analyzed using descriptive statistical tests, number, percentage, mean, standard deviation, median, minimum, and maximum values. Normal distribution of the data was checked for each variable. Correlation coefficients were calculated to determine the relationships between variables. In addition, cause and effect relationships between variables were explained by linear regression analysis. In all calculations and interpretations, the statistical significance level was considered as *p* < 0.05.

## Results

Statistics on the socio-demographic characteristics of mothers and newborns are given in Table 1. The majority of the mothers were between 26 and 34 years of age, 37.5% were university graduates, 60.8% were housewives and 71.7% had health insurance. It was also determined that 85% of the deliveries were performed by cesarean section, 48.3% of the newborns were born prematurely. And fear and anxiety due to the condition of their children were experienced by 51.7% of the mothers.

**Table 1:** The socio-demographic characteristics of the mothers and the newborn (*N* = 120)

Variables	*N*	%
*Age*		
≤ 25	17	21.3
26–29	28	35.0
30–34	19	23.7
≥ 35	16	20.0
*Education Level of the Mother*		
Primary education	33	27.5
High school	42	35.0
University	45	37.5
*Occupation of the mother*		
Working	47	39.2
Housewives	73	60.8
*Health insurance*		
Yes	86	71.7
No	34	28.3
*Economical situation*		
Bad	13	10.8
Middle	41	34.2
Good	66	55.0
*Place of residence*		
Village	16	13.3
District	50	41.7
Province	54	45.0
*Abortus survival*		
Yes	40	33.3
No	80	66.7
*The person who will help you in the family*		
There is	75	91.3
None	45	8.7
*Experiencing a stressful event in the last 1 year*		
Yes	24	20.0
No	96	80.0
*Planned pregnancy*		
Yes	99	82.5
No	21	17.5
*Mode of delivery*		
Normal birth	18	15.0
Cesarean section	102	85.0
*Gender of the Newborn*		
Girl	50	41.7
Boy	70	58.3
*Diagnosis of the baby*		
Prematurity	58	48.3
Transient Tachypnoea of the Newborn	33	27.5
Sepsis	7	5.8
Hyperbilirubinaemia/malnutrition	4	3.3
Other	18	15.0
*Baby’s feeding style*		
Suckling mummy	22	18.3
Parenteral nutrition (TPN)	14	11.7
Enteral feeding (nasogastric/oragastric)	54	45.0
Breast milk and formula are mixed	30	25.0
*Emotions experienced by the mother because of her child’s*		
Fear-anxiety	62	51.7
Hopelessness	5	4.2
Guilt	8	6.7
Rebel	1	0.8
All	27	22.5
None	17	14.2
*Spiritual changes experienced by the mother due to her child’s*		
Increase in worship	6	5.0
Increasing spiritual strength	49	40.8
Decreased spiritual strength	4	3.3
Increase in worship and increase in spiritual power	40	33.3
None	21	17.5
Total	120	100.0
Mother Age (years)^a^	29.63 ± 5.80	(18–47)
Duration of marriage (years)	6.20 ± 5.08	(1–22)
Number of pregnancies	2.30 ± 1.15	(1–7)
Gestational age (weeks)^a^	34.55 ± 4.21	(25–42)
Birth Weight (grams)^a^	2.531,92 ± 1.038	(0.685–5.090)
Birth Height (cm)	45.33 ± 6.15	(26–54)
Length of stay in NICU (days)	8.33 ± 7.94	(7–46)
Frequency of visiting the baby in the NICU	2.11 ± 1.04	(1–4)

^a^Mean ± SD

The mean scores of SOS, RCSS, PSS:NICU and PBS are presented in Table 2. The mean score for SOS was 93.90 ± 15.48. The mean score for RCSS Positive Religious Coping Styles sub-factor was 24.07 ± 3.40 and for Negative Religious Coping Styles sub-factor was 6.14 ± 2.86. Among the PSS:NICU sub-factors, the mean score for images and sounds was 2.88 ± 0.75, the mean score for infant appearance and behavior was 5.82 ± 0.86, and the mean score for parental role change was 4.20 ± 0.60. The mean PSS:NICU total score was found to be 3.84 ± 0.58. For PBS, the mean total score was 71.12 ± 9.04.

**Table 2:** Spiritual Orientation Scale (SOS), Religious Coping Styles Scale (RCSS), Parental Stressor Scale: Neonatal Intensive Care Unit (PSS:NICU) and Parent Belief Scale (PBS) scores (N = 120)

	Minimum	Maximum	Mean	Standard deviation
Spiritual Orientation Scale (SOS)	16.00	112.00	93.30	15.48
*Religious Coping Styles Scale (RCSS)*				
Positive Religious Coping Styles	14.00	28.00	24.07	3.40
Negative Religious Coping Styles	3.00	12.00	6.14	2.86
*Parental Stressor Scale: Neonatal Intensive Care Unit (PSS:NICU)*				
PSS:NICU-Sights and sounds	0.86	4.33	2.88	0.75
PSS:NICU-Infant’s appearance and behaviors	1.45	5.82	3.48	0.86
PSS:NICU-Parental role alteration	2.00	5.71	4.20	0.60
PSS:NICU-Total score	1.74	5.29	3.84	0.58
Parent Belief Scale (PBS)	47.00	90.00	71.12	9.04

The relationships between SOS, RCSS, PSS: NICU and PBS are presented in Table 3. A high level of positive correlation was found between SOS and Positive Religious Coping Styles (*r* = 0.639, *p* < 0.001), and a weak positive correlation was found between SOS and infant appearance and behaviors (*r* = 0.247, *p* = 0.007), parental role change (*r* = 0.205, *p* = 0.024), PSS:NICU (r = 0.199, *p* = 0.029) and PBS (r = 0.351, *p* < 0.001).

**Table 3:** Pearson correlation coefficient between mothers’ Spiritual Orientation Scale (SOS), Religious Coping Styles Scale (RCSS), Parental Stressor Scale: Neonatal Intensive Care Unit (PSS:NICU), and Parent Belief Scale (PBS)

Variable	(1)	(2)	(3)	(4)	(5)	(6)	(7)	(8)
(1) SOS	1							
(2) Positive Religious Coping Styles	*r*: 0.639 *p*: 0.000*	1						
(3) Negative Religious Coping Styles	*r*: 0.085 *p*: 0.359	*r*: 0.062 *p*: 0.503	1					
(4) PSS:NICU Sights and sounds	*r*: − 0.031 *p*: 0.734	*r*: − 0.042 *p*: 0.645	*r*: − 0.082 *p*: 0.373	1				
(5) PSS:NICU Infant’s appearance and behaviors	*r*: 0.247 *p*: 0.007*	*r*: 0.133 *p*: 0.149	*r*: -0.085 *p*: 0.358	*r*: 0.468 *p*:0.000*	1			
(6) PSS:NICU Parental role alteration	*r*: 0.205 *p*: 0.024*	*r*: 0.163 *p*: 0.075	*r*: − 0.048 *p*: 0.600	*r*: 0.397 *p*:0.000*	*r*: 0.397 *p*:0.000*	1		
(7) PSS:NICU Total score	*r*: 0.199 *p*: 0.029*	*r*: 0.144 *p*: 0.117	*r*: -0.088 *p*: 0.339	*r*: 0.683 *p*:0.000*	*r*: 0.805 *p*:0.000*	*r*: 0.826 *p*:0.000*	1	
(8) PBS	*r*: 0.351 *p*: 0.000*	*r*: 0.256 *p*: 0.005*	*r*: 0.148 *p*: 0.107	*r*: − 0.038 *p*: 0.678	*r*: 0.093 *p*: 0.314	*r*: 0.055 *p*:0.550	*r*: 0.064 *p*:0.485	*1*

^*^p < 0.05

There was no statistically significant correlation (*p* = 0.232, *p* > 0.05) for SOS total score PSS:NICU. However, a significant correlation was found between SOS and PBS (*β* = 0.364; *t* = 4.251; *p* < 0.001). As the SOS Total score increased, the PBS score also increased. The rate of SOS Total explaining PBS was 13% (*R*^2^ = 0.133). There is no significant correlation between Positive Religious Coping Styles and PBS:NICU (*p* = 0.117, *p* > 0.05). However, there is a significant correlation between Positive Religious Coping Styles and PBS (*β* = 0.256; *t *= 2.873; *p* = 0.005, *p* < 0.05). As the Positive Religious Coping Styles score increases, the PBS score also increases. The rate of this variable explaining PBS is 6% (*R*^2^ = 0.065). There is no significant correlation between Negative Religious Coping Styles and PBS:NICU (*p* = 0.339, *p* > 0.05). Similarly, there is no significant correlation between Negative Religious Coping Styles and PBS (*p* = 0.107, *p* > 0.05) (Table 4).

**Table 4:** Linear regression analysis for effect of Spiritual Orientation Scale (SOS) and Religious Coping Styles Scale (RCSS) scores on Parental Stressor Scale: Neonatal Intensive Care Unit (PSS:NICU) and Parent Belief Scale (PBS) scores

PSS:NICU*	*β*_0_ (95% CI)	S. error	*β*_1_	*t*	*p*
Fixed	117.483 (95.182–139.784)	11.262		10.432	0.000
SOS total	0.140 (-0.091–0.371)	0.117	0.110	1.202	0.232
PBS**	*β*_0_ (95% CI)	S. error	*β*_1_	*t*	*p*
Fixed	50.840 (41.268–60.412)	4.834		10.518	0.000
SOS total	0.213 (0.114–0.312)	0.050	0.364	4.251	0.000
PSS:NICU***	*β*_0_ (95% CI)	S. error	*β*_1_	*t*	*p*
Fixed	3.258 (2.510–4.005)	0.378		8.629	0.000
Positive Religious Coping Styles	0.025 (− 0.006–0.055)	0.016	0.144	1.580	0.117
PBS*****	*β*_0_ (95% CI)	S. error	*β*_1_	*t*	*p*
Fixed	54.784 (43.409–66.160)	5.745		9.537	0.000
Positive Religious Coping Styles	0.679 (0.211–1.147)	0.236	0.256	2.873	0.005
PSS:NICU****	*β*_0_ (95% CI)	S. error	*β*_1_	*t*	*p*
Fixed	3.958 (3.709–4.207)	0.126		31.453	0.000
Negative Religious Coping Styles	− 0.018 (− 0.055–0.019)	0.019	− 0.088	− 0.960	0.339
PBS******	β_0_ (95% CI)	S. error	*β*_1_	*t*	*p*
Fixed	68.258 (64.40–72.11)	1.946		35.076	0.000
Negative Religious Coping Styles	0.467 (− 0.102–1.036)	0.287	0.148	1.624	0.107

^*^Dependent variable: PSS:NICU *F* = 1.444, *p* = 0.232, *R* = 0.110, *R*^2^ = 0.012, *β*0 unstandardized beta coefficient, *β*1 standardized beta coefficient

^**^Dependent variable: PBS *F* = 18.072, *p* = 0.000, *R* = 0.364, *R*^2^ = 0.133, *β*0 unstandardized beta coefficient, *β*1 standardized beta coefficient,

^***^Dependent variable: PSS:NICU *F* = 2.497, *p* = 0.117, *R* = 0.144, *R*^2^ = 0.021, *β*0: unstandardized beta coefficient, *β*1: standardized beta coefficient

^****^Dependent variable: PBS *F* = 8.252, *p* = 0.005, *R* = 0.256, *R*^2^ = 0.065, *β*0 unstandardized beta coefficient, *β*1 standardized beta coefficient

^*****^Dependent variable: PSS:NICU *F* = 0.922, *p* = 0.339, *R* = 0.088, *R*^2^ = 0.008, *β*0 unstandardized beta coefficient, *β*1 standardized beta coefficient

^******^Dependent variable: PBS *F* = 2.639, *p* = 0.107, *R* = 0.148, *R*^2^ = 0.022, *β*0 unstandardized beta coefficient, *β*1 standardized beta coefficient

## Discussion

The neonatal period covers the first 28 days of life, when infant mortality is most common. Hospitalization of their babies in the neonatal intensive care unit is a great source of stress for families who expect to have a healthy baby in this period. In the past, many studies have been conducted to measure the stress level of parents whose babies are hospitalized in the NICU. The present study revealed the results of the relationship between spiritual orientation and religious coping styles, stress and NICU parental beliefs of mothers whose babies are hospitalized in NICU. Pichler-Stachl et al. (2019) measured the stress level of parents whose infants were hospitalized in the intensive care unit in their study on 47 parents; when the scores they received from the ‘Images and Sounds’ group at admission to the intensive care unit were compared, an average of 1.86 ± 0.79 was found in mothers and 1.74 ± 0.77 in fathers. As a result of the study, it was found that the stress level of mothers was higher than that of fathers; it was observed that the age of the mother was directly proportional to the stress level, whereas there was no relationship between the age and stress level of fathers (Pichler-Stachl et al., 2019). In the study, mothers’ spiritual orientation was found to be highly positively associated with positive religious coping styles and weakly positively associated with infant appearance and behaviors, parental role change, parental stress and parental beliefs. This indicated that spiritual orientation had a link with parental stress and beliefs in general, but this link was not strong. In Çekin and Turan’s (2018) study, when the mean total stress scores were examined, there was no significant difference between the total stress scores of mothers and fathers (*p* > 0.05). However, it was observed that the mean stress scores of mothers were higher than the mean stress scores of fathers. It was observed that the stress level in the parents of infants who needed respiratory support (126.48 ± 23.73) was higher than the parents of infants who did not need respiratory support (108.60 ± 32.46) (Çekin & Turan, 2018). In our study, the mean stress level of the parents was calculated as 2.88 and it was found that parental stress in NICU was generally at a moderate level. The results of the study showed that there was a significant positive correlation between PSS:NICU and infant appearance and behavior (*r* = 0.247, *p* = 0.007). This finding suggests that parental stress in the NICU is associated with the infant’s physical condition and behavioral responses. In the intensive care setting, the infant’s health and behaviors are among the important factors affecting parental stress levels. This relationship suggests that parents experience increased anxiety and stress as they observe their infant’s condition.

Studies have been conducted comparing different methods to determine the causes of stress in parents and to reduce high stress levels. In one study, it was observed that 91.4% of the parents wanted to ask questions and get information about the condition of their babies, 75.3% tried to get information from different sources, 87.7% tried to reduce stress by sharing it with their spouses and 92.6% prayed for their babies (Miles et al., 1993). In similar studies, informing parents in detail and introducing them to health care providers (Turan & Basbakkal, 2006), avoiding the use of medical terms while informing parents (Erdeve et al., 2008), supporting parental participation in care (Miles et al., 1993), and practicing kangaroo care (Melnyk et al., 2014) were listed as methods that reduce the stress level of mothers. Recent studies also reveal the effect of spirituality and religious orientations on coping with stress. Parents believe that their belief in supernatural powers has a protective and healing power over their children and that their children are safer when they pray or perform various rituals (Sadeghi et al., 2016; Sekhavatpour et al., 2020). It is known that spiritual needs are not limited to religious beliefs and rituals. In his hierarchy of needs, Maslow defined primary needs as food, drink, clothing, shelter, sleep and sexual activity, and secondary needs as security, companionship, acceptance, belonging and self-actualization. Therefore, in addition to their divine beliefs and needs, parents also express needs such as hope, compassion, peace and understanding (Büssing et al., 2018). Sadeghi et al. (2016) evaluated the spiritual needs of parents during the mourning process in semi-structured interviews with 24 families experiencing infant loss and grief process in a neonatal intensive care unit in Iran. As a result of the study, belief in a supernatural power was the most important spiritual need identified for the participating families. Most of the families saw their faith and religious rituals such as prayers, donations, reading the Qur’an, and meeting with religious officials as a source of strength in the face of critical situations (Sadeghi et al., 2016). Ercan, Kırlıoğlu, & Kalaycı Kırlıoğlu (2019) investigated the acceptance processes and coping methods of parents with children with special needs and found that this process is an exhausting and consuming process for parents and that parents are positively affected by religious coping methods in this process (Ercan, Kırlıoğlu, & Kalaycı Kırlıoğlu, 2019). In our study, the average of positive religious coping styles was higher than the negative ones, indicating that parents adopted more positive religious coping methods.

## Strengths and Limitations

This study involves some limitations. The relational design of the study could not determine causality. In this sense, there is a need for longitudinal studies to be performed in the future. Another limitation is that the findings are based on mothers’ self-report. In addition, the large number of items on the scale is another limitation. Longitudinal studies with larger and more diverse samples are recommended to address these limitations.

## Conclusion

The results of this study reveal the impact of socio-demographic characteristics of mothers and newborns on parents’ spiritual dispositions and coping methods. It was found that the majority of mothers had a strong spiritual disposition and positive religious coping methods, and these characteristics played a supportive role in coping with the stress experienced in the NICU. These findings suggest that parents’ strong levels of faith help them manage their anxiety about their newborns’ health status. In this context, it was concluded that strong spiritual dispositions and positive religious coping strategies can increase parents’ psychological resilience in the health and care process. Based on these results, it can be recommended to have information about the factors affecting the spiritual well-being levels of mothers and fathers whose babies are hospitalized in the NICU. In addition, spiritual beliefs and practices of mothers and fathers should be accepted, supported and appropriate environments should be provided for this.

## Data Availability

The data sets generated during and/or analyzed during the current study are not publicly available, but are available from the corresponding author on reasonable request.

## Appendix 1

Instrument for data collection: Spiritual Orientation Scale.

**Table:**

	Spiritual orientation scale
1	I feel the presence of a higher power in the depths of my soul
2	I have spiritual experiences that give me peace
3	I feel loved by a divine being
4	Only an infinite being can understand man at the ultimate point
5	Prayer/meditation is an important part of my spiritual life
6	My belief in a divine power helps me to cope with difficulties in life
7	If one really searches for the meaning of one’s life, one can find answers
8	My belief in a divine being influences my behavior
9	By praying, I can feel closer to what I believe in
10	My belief in a divine being gives meaning to my life
11	Prayer/meditation provides me with emotional support
12	My communication with the spiritual dimension is good for my mental health
13	I feel protected by a higher power
14	I experience a sense of wholeness in the serenity of prayer/meditation
15	Admiring nature strengthens my spiritual feelings
16	My faith strengthens my communication with the people around me

## Appendix 2

Instrument for data collection: NICU-Parental Beliefs Scale.

**Table:**

	NICU parental beliefs scale
1	I know what characteristics and behaviors are common in premature babies hospitalized in the NICU
2	I am sure that what I do for my baby will be what is best to help him/her deal with being in the NICU
3	I feel comfortable in caring for my baby in the NICU
4	I know what characteristics and behaviors to expect in my baby while he/she is in the NICU
5	I am sure about what things I can do to best help my baby get through the NICU experience
6	I am sure that I can meet my baby’s emotional needs while he/she is in the NICU
7	I know why my baby has the characteristics and behaviors that he/she does in the NICU
8	I feel confident in telling the nurses and doctors about what will best help my baby while he/she is in the NICU
9	I am clear about how to help take care of my baby in the NICU
10	I know how my baby will probably respond to me while he/she is in the NICU
11	I am sure about how my emotions will affect my baby while he/she is in the hospital
12	I am clear about how my baby will react when he or she is getting too much stimulation in the NICU
13	I am sure about the things that I can do to make my baby feel most secure while he/she is in the NICU
14	I know how my baby’s appearance and behaviors are different from a full-term baby’s appearance and behaviors
15	I know the best times to communicate with or interact with my baby
16	I am confident in asking the doctors and nurses questions about my baby’s medical condition
17	I know what my baby will do when he or she is stressed
18	I am clear about what my baby will look or act like when he or she is ready to communicate with me
